# Ecotoxicological Impact of the Bioherbicide Leptospermone on the Microbial Community of Two Arable Soils

**DOI:** 10.3389/fmicb.2016.00775

**Published:** 2016-05-24

**Authors:** Sana Romdhane, Marion Devers-Lamrani, Lise Barthelmebs, Christophe Calvayrac, Cédric Bertrand, Jean-François Cooper, Franck E. Dayan, Fabrice Martin-Laurent

**Affiliations:** ^1^Biocapteurs Analyse Environnement, EA 4218, University of Perpignan via DomitiaPerpignan, France; ^2^Centre de Recherches Insulaires et Observatoire de l'Environnement, USR 3278 EPHE-Centre National de la Recherche Scientifique, University of Perpignan via DomitiaPerpignan, France; ^3^Institut National de la Recherche Agronomique, UMR 1347 Agroécologie, EcolDurDijon, France; ^4^Bioagricultural Sciences and Pest Management Department, Colorado State UniversityFort Collins, CO, USA

**Keywords:** bacterial community, microbial ecotoxicology, biodegradation, bioherbicide, leptospermone

## Abstract

The ecotoxicological impact of leptospermone, a β-triketone bioherbicide, on the bacterial community of two arable soils was investigated. Soil microcosms were exposed to 0 × (control), 1 × or 10 × recommended dose of leptospermone. The β-triketone was moderately adsorbed to both soils (i.e.,: *K*_fa_ ~ 1.2 and *K*_oc_ ~ 140 mL g^−1^). Its dissipation was lower in sterilized than in unsterilized soils suggesting that it was mainly influenced by biotic factors. Within 45 days, leptospermone disappeared almost entirely from one of the two soils (i.e., DT_50_ < 10 days), while 25% remained in the other. The composition of the microbial community assessed by qPCR targeting 11 microbial groups was found to be significantly modified in soil microcosms exposed to leptospermone. Pyrosequencing of 16S rRNA gene amplicons showed a shift in the bacterial community structure and a significant impact of leptospermone on the diversity of the soil bacterial community. Changes in the composition, and in the α- and β-diversity of microbial community were transient in the soil able to fully dissipate the leptospermone, but were persistent in the soil where β-triketone remained. To conclude the bacterial community of the two soils was sensitive to leptospermone and its resilience was observed only when leptospermone was fully dissipated.

## Introduction

Agriculture is facing critical challenges to ensure high crop production and quality, while preserving environmental and human health (Brussaard et al., 2010). Among pressures exerted by agriculture on the environment, synthetic plant protection products (PPPs) are of concern because they can persist in arable soils and be transferred to different compartments of the environment where they are frequently detected (Margni et al., 2002; Polyrakis, 2009). In this context, the use of natural active compounds, also known as biopesticides, may be an interesting alternative for crop protection as they are considered to be less harmful and environmentally safer (Dewhurst, 2001; Dayan et al., 2009, 2011; Cantrell et al., 2012; Seiber et al., 2014). Within the last few years, several studies highlighted the effectiveness of biopesticides for the control of different pests in various crops (Copping and Menn, 2000). Due to the diversity of biopesticide modes of action, risks of resistance emergence is considerably reduced (Copping and Menn, 2000; Dayan and Duke, 2014). Consequently, the biopesticide market has been gaining greater approval and interest for pest management (Copping and Menn, 2000; Cantrell et al., 2012; Seiber et al., 2014).

Despite this enthusiasm for natural alternatives, few bioherbicides are commercially available; among them manuka oil is being considered as a pre- and post-emergence herbicide to control several broadleaf and grass weeds at a rate of 3 L ha^−1^ (Dayan et al., 2011). The active ingredient in the essential oil of Manuka tree (*Leptospermum scoparium*) is the β-triketone leptospermone [2,2,4,4-tetramethyl-6-(3-methyl-1-oxobutyl)-1,3,5-cyclohexanetrione (Hellyer, 1968; Dayan et al., 2007, 2011). This natural herbicide is also produced by other species such as the allopathic plant bottlebrush plant (*Callistemon citrinus*) (Gray et al., 1980; Mitchell et al., 2001). Leptospermone targets 4-hydroxyphenylpyruvate dioxygenase (HPPD), a key enzyme in plant carotenoid biosynthesis, that catalyzes the conversion of 4-hydroxyphenylpyruvate into homogentisate leading to foliage bleaching of treated weeds (Schulz et al., 1993; Meazza et al., 2002; Dayan et al., 2007, 2011). HPPD is also the target site for commercial synthetic β-triketones herbicides (e.g., sulcotrione, mesotrione, and tembotrione), which were derived from the structural backbone of leptospermone (Gray et al., 1980; Schulz et al., 1993; Lee et al., 2008). HPPD is expressed by many organisms, including plants (Moran, 2005), and several bacteria (Lee et al., 2008), that could be indirectly affected by this family of herbicides. Indeed, this bioherbicide also has antimicrobial, antiviral, and acaricidal activities (Jeong et al., 2009a,b).

To date there are only few studies describing the environmental fate and ecotoxicological impact of biopesticides on non-target organisms (NTO). In spite of their natural origin, biopesticides are active compounds used to eradicate target organisms in crops. Like other agrochemicals, once released in the soil, they may undergo a range of abiotic and biotic processes (i.e., sorption, degradation, and transport) modulating their environmental fate and conditioning their side effects on NTO. For example, azadirachtin, an natural insecticide from neem (*Azadirachta indica*), exerts adverse effect on soil microbial community (Gopal et al., 2007; Gupta et al., 2013; Singh et al., 2015a,b). These reports underscored the need to assess the environmental fate and ecotoxicological impact of biopesticides on NTO. Among NTOs, the European Food Safety Authorithy (EFSA) proposed soil microorganisms, which support numerous functions (Whitman et al., 1998; Nannipieri et al., 2003; Philippot et al., 2012) contributing to regulation and maintenance of soil ecosystemic services, as specific protection goals (SPGs) for environmental risk assessment of PPPs (EFSA, 2010; Science for Environment Policy, 2015). Consequently, the ecotoxicological impact of PPPs on soil microorganisms will have to be assessed to fulfill the SPGs. Within this context, innovative tools relying on high throughput soil DNA analyses give new insight for assessing ecotoxicological impact of PPPs on soil microbial community (Martin-Laurent et al., 2013; Cai et al., 2015; Wang et al., 2015).

Although leptospermone is considered as a selective and efficient bioherbicide candidate for crop protection (Dayan et al., 2009, 2011; Cantrell et al., 2012), only few studies have examined its environmental fate. It has a relatively short half-life in water and is rapidly dissipated *via* photodegradation (Trivella et al., 2015). However, there is no report on abiotic and biotic processes contributing to leptospermone dissipation. Likewise its ecotoxicological impact on soil microbial community has not been assessed although a vast majority of microorganisms harbor *hppd* gene, and could therefore be potentially sensitive to leptospermone. To tackle these questions, this work reports a microcosm study designed to describe the fate of leptospermone and its ecotoxicological impact on the composition and the diversity of the soil microbial community. Two agricultural soils with contrasted physicochemical properties were selected as model environments. Leptospermone was applied at 1 ×, or 10 × recommended agronomical rate on soil microcosms along with an untreated control. The scenario of exposure of the microbial community to leptospermone was estimated by monitoring its dissipation in soil microcosms. The ecotoxicological impact of leptospermone on the soil bacterial composition and diversity was investigated from DNA samples, extracted directly from soils, by means of real-time quantitative PCR targeting eleven microbial groups and pyrosequencing of 16S rRNA gene amplicons, respectively.

## Materials and methods

### Soil sampling and characteristics

Soil samples were collected from the surface layer (0–20 cm) of two different arable field sites [Perpignan (P) and Saint Jean de Fos (SJF), France] selected according to their physicochemical properties. P soil is an experimental field site having a β-triketone history treatment (Calvayrac et al., 2012) and SJF soil was neither cultivated nor treated with pesticides for the last 5 years. Soil samples were sieved to 2 mm and soil moisture was measured. Soils were stored at 4°C until use. The composition and characteristics of the P soil was 13.9% clay, 60.5% silt, 25.6% sand, 20% soil humidity, 1.7% organic matter, 0.98% organic carbon, 15.5 meq 100 g^−1^ cation exchange capacity (CEC), 214% Ca^2+^/CEC, and pH in water 8.1. The composition and characteristics of the SJF soil was 25.8% clay, 27.3% silt, 46.9% sand, 15% soil humidity, 1.5% organic matter, 0.9% organic carbon, 10.4 meq 100 g^−1^ cation exchange capacity (CEC), 98% Ca^2+^/CEC, and pH in water 7.62.

### Microcosm set up

Pure leptospermone was synthesized as described by Owens et al. (2013). Soil samples (20 g) were treated with a leptospermone solution prepared in methanol at 0 × (D0, control), 1 × (D1, 5 μg g^−1^), and 10 × (D10, 50 μg g^−1^) recommended field dose. Methanol was then evaporated and the microcosms were moistened to reach 33% of soil water-holding capacity and incubated in the dark for 45 days at 22°C. The abiotic degradation of leptospermone in the microcosms was assessed in soils sterilized using γ-radiation. Soil samples were analyzed at 0, 2, 4, 8, 15, 30, and 45 days after the treatment (*d*). Soil microcosms were prepared in triplicates for each soil, treatment and time points (*n*_tot_ = 252).

### Fate of leptospermone in soil microcosms

#### Kinetics of dissipation

During the time course of the incubation, the dissipation of leptospermone was measured. At each sampling time, P and SJF soil samples (10 g) were acidified by 3 mL of 0.1 M hydrochloric acid solution and homogenized by vortex mixing, then extracted twice with 30 mL of ethyl acetate for 60 min. Organic phases were filtered on a Whatman filter GF/A and evaporated to dryness at 30°C. Dry extracts were eluted with methanol (5 mL). The final extract was analyzed by high-performance liquid chromatography (HPLC) Jasco apparatus equipped with a Phenomenex Luna C18 column (150 × 3.0 mm; 5 μm) and a diode array detector Jasco 875-UV set. The mobile phase consisted of a mixture of water (A) and acetonitrile (B) both acidified by 0.1% formic acid and delivered at a flow rate of 0.5 mL min^−1^. The gradient method was: for 3 min 95% (A—5% (B), from 3 to 15 min 5% (A)—95% (B) maintained for 7 min, then back to 95% (A)—5% (B) from 22 to 26 min. No chromatographic interference was detected. Detection was carried out by using UV light (wavelength, 280 nm). The quantification limit of the analysis method was estimated at 0.2 mg L^−1^. Calibration curves were established using blank soil samples prepared according to the above procedures and spiked with a concentration range of leptospermone from 0.5 to 50 μg g^−1^. The obtained curves (*R*^2^ = 0.99, *n* = 3) were used to determine concentrations of remaining leptospermone in soil extracts.

#### Adsorption isotherms

Adsorption isotherms of leptospermone to P and SJF soils were measured by batch equilibrium method (Wilson and Foy, 1992; OECD, 2000; Cherrier et al., 2004). In order to determine the time required to reach the equilibrium, 5 mL of leptospermone at 20 mg L^−1^ prepared in 0.01 M CaCl_2_ solution were added to 1 g of each soil, placed on a vertical shaker and agitated for 0.5, 1, 2, 3, 4, 8, and 24 h. Soil suspensions were centrifuged at 3500 × g for 10 min. The supernatants were recovered and the pH was adjusted to pH 9 with 1 M Tris buffer. Leptospermone remaining in supernatants was analyzed by HPLC/UV. Adsorption isotherms were obtained from HPLC analysis of soil samples spiked with a range of leptospermone solutions prepared at different concentrations (1, 2, 5, 10, 20, and 40 mg L^−1^) and agitated for three h following the protocol described above.

### Ecotoxicological impact of leptospermone on soil bacterial community

#### Direct soil DNA extraction

DNA was extracted from 1 g of each soil sample according to the ISO 11063 standard derived from Martin-Laurent et al. (2001). Briefly, samples were mixed with 4 mL of extraction buffer containing 100 mM EDTA, 100 mM Tris (pH 8.0), 100 mM NaCl, 1% (w/v) polyvinylpyrrolidone, and 2% (w/v) sodium dodecyl sulfate in a 5 mL mini-bead-beater tube containing 2 g of 106-μm-diameter glass beads and 8 glass beads of 2-mm-diameter. Samples were then homogenized for 30 s at 1600 rpm in a mini-bead beater cell disrupter (Mikro-Dismembrator©, Sartorius AG, Germany). After an incubation of 15 min at 70°C, soil components were eliminated by centrifugation at 14000 × g for 1 min at 4°C. Proteins were then precipitated with sodium acetate at a final concentration of 0.3 M. After centrifugation at 14000 × g for 10 min, supernatants were recovered and nucleic acids were precipitated with ice-cold isopropanol and washed with 70% ethanol. DNA was purified using a Sepharose 4B spin, polyvinylpyrrolidone (PVPP) spin columns and NucleoSpin® kits (Machery-Nagel, Germany). DNA concentration was quantified using the Quant-iT™ PicoGreen® dsDNA Assay Kit (Invitrogen™, France) following the manufacturer's instructions. The absence of PCR inhibitors in DNA extracts was verified according to Henry et al. (2006). No inhibition was detected in 2 μL soil DNA template diluted at 0.1 ng μL^−1^.

#### Abundance of total bacteria and phylum-specific groups

The abundances of total bacteria, several bacterial taxa and *Crenarchaeota* were measured in P and SJF soils treated (D1, D10) or not (D0) with leptospermone after 0, 2, 4, 8, 15, 30, and 45 days of incubation by real-time quantitative PCR (qPCR) targeting 16S rRNA gene sequences according to ISO/DIS 17601 standard as previously described (Petric et al., 2011). Eleven taxa-specific primers (Supplementary Table 1) were used to quantify α*-Proteobacteria*, β*-Proteobacteria*, γ*-Proteobacteria, Acidobacteria, Actinobacteria, Verrucomicrobia, Bacteroidetes, Firmicutes, Gemmatimonadetes, Planctomycetes*, and *Crenarchaeota* (Muyzer et al., 1993; Ochsenreiter et al., 2003; Fierer et al., 2005; Muhling et al., 2008; Philippot et al., 2009). qPCR assays were carried out in an ABI 7900 HT Real-time PCR System (Applied Biosystems, USA) in a final reaction mixture of 15 μL containing SYBR green PCR Master Mix (Absolute™ SYBR® Green Rox Abgene, Thermo Fisher Scientific, France), 250 ng of T4 gp32 (Qbiogene, MP Biomedicals, France), 1 mM of each primer and 0.2 ng of soil DNA template. qPCR runs were as follows: denaturation step for 15 min at 95°C, then 35 cycles of 15 s of denaturation at 95°C, 30 s of annealing at the optimal temperature for primer annealing, elongation for 30 s at 72°C, 30 s for data acquisition at 80°C, and a melting curve stage with 15 s at 95°C, 15 s at 78°C, and 15 s at 95°C. Standard curves were generated using serial dilutions of linearized plasmid pGEM-T containing each standard gene sequence (ranging from 10^7^ to 10^2^ copies per qPCR reaction). For each qPCR assay, qPCR calibration is performed in triplicate and three no-template controls (NTC) were also included.

#### Diversity of the bacterial community

The diversity of the bacterial community was measured for P and SJF soils treated (D1 or D10) or not with leptospermone for 4 and 45 days. Diversity was monitored by 454 pyrosequencing of the V4 ultravariable region of 16S rRNA gene using 515F (5- GTGCCAGCMGCCGCGGTAA- 3) and 806R (5- GGACTACHVGGGTWTCTAAT-3) primer pair (Turner et al., 1999; Liu et al., 2007; Caporaso et al., 2011). A two-step PCR procedure was used to amplify 16S rRNA gene from obtained DNA extracts as described by Berry et al. (2012). In the first step of this protocol, 20 cycles of amplification were performed in triplicate using the 515F-806R primer pair. Triplicates of each PCR reaction were pooled to a one PCR product. Then, 1 μL of these 16S rRNA amplicons was used as template to carry out 15 cycles of amplification with barcoded primers. The size of the amplicons was verified by electrophoresis performed on a 1.5% agarose gel. 16S rRNA amplicons were pooled and purified using the QIAEX II kit (Qiagen, France). Each sample was quantified using the Quant-iT™ PicoGreen® dsDNA Assay Kit (Invitrogen, France). Pooled samples were sequenced on a 454 FLX Genome Sequencer (Roche) by Genoscreen (Lille, France) using the Titanium Chemistry. Sequence data analysis was performed using QIIME (Quantitative Insights into Microbial Ecology) version 1.8.0 (Caporaso et al., 2010b). After splitting the libraries, 16S rRNA sequences were denoised, chimera were removed and Operational Taxonomic Units (OTUs) were selected at 97% similarity using UPARSE (Edgar, 2010). Representative 16S rRNA sequences for each OTU were aligned using Pynast (Caporaso et al., 2010a) and their taxonomy was assigned by comparison with the GreenGenes database (http://www.ncbi.nlm.nih.gov/genbank/) using uclust algorithm (Edgar, 2010). Maximum likelihood phylogeny was calculated using FastTree 2 algorithm (Price et al., 2010). Phylogenetic tree plotting with additional OTU abundance data was performed using the Interactive Tree of Life (iTOL) webserver (Letunic and Bork, 2007). Two hundred and fifty OTUs representing 65% of the total number of sequences are represented on this phylogenetic tree. For each treatment, the average abundance of each OTU was calculated among replicates. Several α-diversity indices were calculated using the rarefied OTU table at the depth of 1900 sequences per sample (PD whole tree, Chao 1, Dominance, Simpson, and Shannon indices). Principal Coordinate Analysis (PCoA) plots were generated in QIIME based on weighted UniFrac distance matrix and coordinates were used to draw 3D figures. Sequences were deposited in GenBank to the sequence read archive (SRA) under the accession number SRP061834.

### Statistical analysis

Data were subjected to statistical analysis of variance with a significance threshold set at a *p*-value of 0.05 using R software (R foundation for statistical computing, Austria). For qPCR results, abundances of specific taxa were log-transformed and subjected to an analysis of variance (ANOVA). The homogeneity of variances was verified and Tukey's test was performed for each treatment and each time point. Not all qPCR data showed a normal distribution (16S rRNA, α*-Proteobacteria*, β*-Proteobacteria*, γ*-Proteobacteria, Bacteroidetes, Firmicutes*, and *Crenarchaeota*). Consequently, non-parametric test (Kruskal–Wallis test and Mann–Whitney U) was carried out on non-normal distributed data and diversity indices to determine differences between treatments. To detect significant differences among treatments and time, a PERMANOVA analysis was carried out on the distance matrix for principal coordinate analysis (PCoA) using the ADONIS function from R package “vegan” (Oksanen et al., 2011). Groups affected by leptospermone treatment were detected by the nearest shrunken centroid approach using the “pamr” package under R (Tibshirani et al., 2002).

## Results

### Fate of leptospermone in soil microcosms

**Figure 1:**
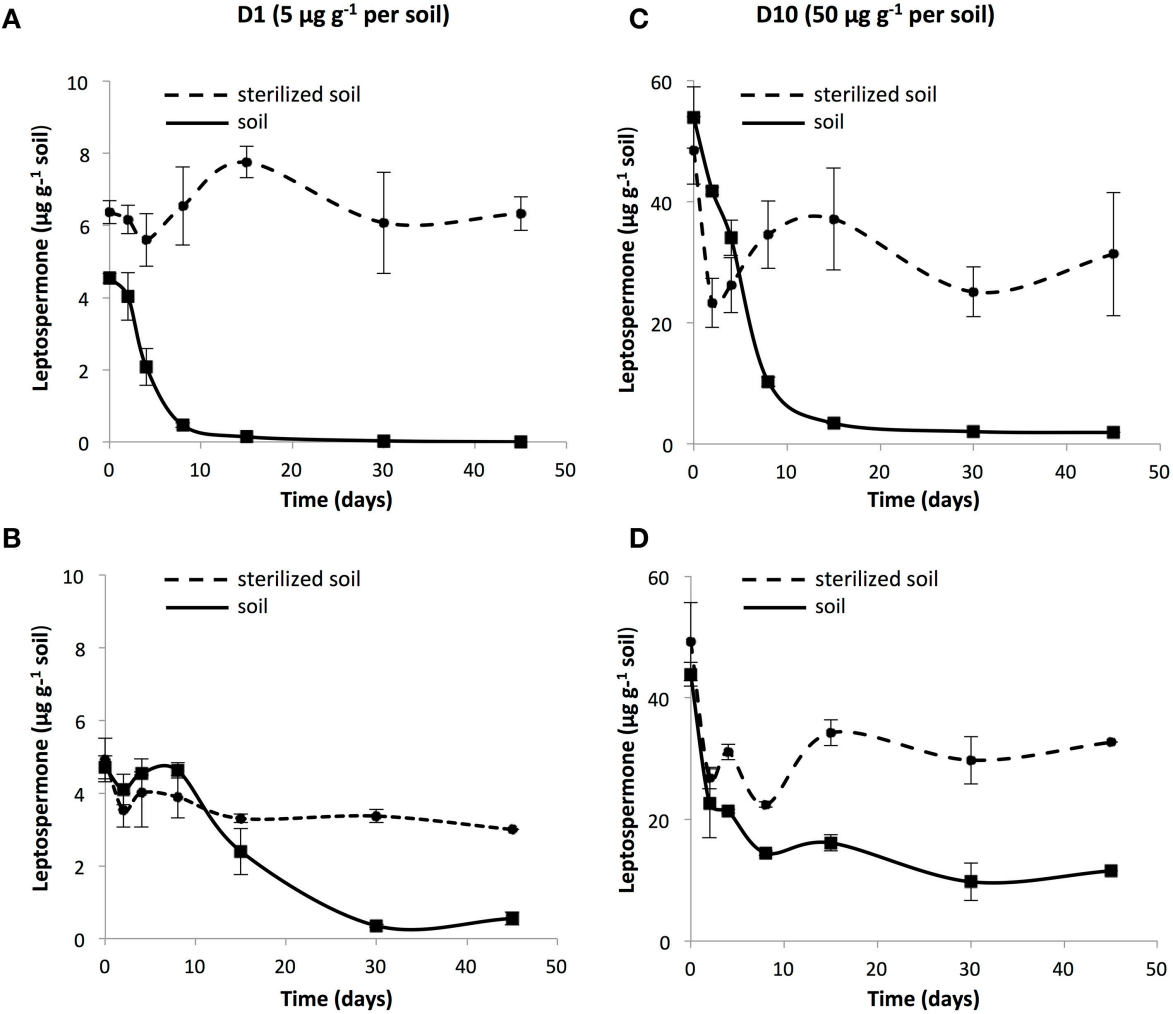
**Dissipation kinetics of 1 × recommended rate leptospermone (D1; 5 μg g^−1^) in sterilized (●) and unsterilized soils (■) from (A) P and (B) SJF, and 10 × the recommended rate (D10; 50 μg g^−1^) in the soil from (C) P and (D) SJF**. Standard deviations are indicated (*n* = 3).

The soil behavior of leptospermone was evaluated with adsorption isotherms in P and SJF soils, and Freundlich relationships were used to evaluate the sorption behavior according to OECD guideline. Equilibrium status was reached after 2 h for both soils. Leptospermone was moderately adsorbed on the two soils. *K*_fa_-values were similar: 1.29 and 1.23 for P and SJF soils, respectively (*R*^2^ >0.96). *K*_oc_-values, representing the adsorption of the pesticide normalized by soil organic carbon content, were slightly higher for P soil (143.86 mL g^−1^) than SJF soil (136.66 mL g^−1^). All along the incubation period, the dissipation of the bioherbicide was monitored in D1 and D10 in P and SJF soils (Figure 1). Leptospermone was extracted with a recovery rate of 73 and 50% for P and SJF soils, respectively. In D1 microcosms, a lag phase of 2.5 and 8 days was observed for P and SJF soils, respectively. In contrast, no lag phase was observed in D10 microcosms for both soils. Leptospermone half-life (DT_50_) in P soil estimated from dissipation kinetics was about 4 and 6 days in D1 and D10 microcosms, respectively. In contrast, in SJF soil leptospermone DT_50_ was slightly longer than those recorded in P soil, averaging 9 days, and was not depending on initial applied concentration. By the end of the incubation period, almost all the leptospermone was dissipated from D1 and D10 P soil while about 25% of leptospermone initially added remained in the D10 SJF soil. A rather low dissipation of leptospermone (5 to 20%) was observed in sterilized soils, suggesting that dissipation was mainly governed by biotic processes.

### Impact of leptospermone on the composition of the microbial community

The impact of leptospermone on the composition of the microbial community in the two soils was monitored by quantifying the abundance of the total bacterial community and 11 microbial groups. Total 16S rRNA gene copy numbers ranged from 7.84 × 10^4^ to 1.55 × 10^7^ sequences per nanograms of DNA were extracted from soil microcosms. Although P and SJF soils had different physicochemical properties, the abundance of the total bacterial community did not differ significantly. No significant time or soil effects were observed on the total bacterial community. A multiple comparison test showed a significant impact of the treatment on the abundance of the total bacterial community at 4 days for P soil (*p* = 0.03, Kruskal–Wallis). For SJF soil, the abundance of bacterial community was fluctuating, however, D0 community did not significantly differ from D1 and D10 whatever the sampling time (data not shown).

The possible impact of leptospermone on the composition of the microbial community was further investigated by quantifying the abundances of eleven taxa (α*-Proteobacteria*, β*-Proteobacteria*, γ*-Proteobacteria, Acidobacteria, Actinobacteria, Verrucomicrobia, Bacteroidetes, Firmicutes, Gemmatimonadetes, Planctomycetes*, and *Crenarchaeota*). For each taxonomic group, qPCR results were expressed as percentage of total microbial community estimated by summing the abundances of all the phyla (Supplementary Figure 1). Before treatment, the microbial community of the two soils was dominated by two phyla (i.e., *Actinobacteria* and *Bacteroidetes*) representing up to 70% of the total bacterial community. Leptospermone treatment modified significantly the abundances of α-, β-, and γ*-Proteobacteria, Acidobacteria, Actinobacteria*, and *Firmicutes* (*p* < 0.001). The abundance of β*-Proteobacteria* increased in the presence of leptospermone at 0, 2, 4, 8, 15, and 45 days in P soil, and at 4, 8, 15, 30, and 45 days in SJF soil (*p* < 0.05). Similarly, the abundance of γ*-Proteobacteria* was significantly increased at the higher leptospermone dose (D10) in P (2 days) and SJF (2, 4, 8, 15, and 30 days) soils. In contrast, the relative abundance of *Acidobacteria* was significantly decreased in P (0, 2, 4, and 30 days) and SJF (8 days) treated soils (*p* < 0.05) as compared to respective D0. Similarly, the relative abundance of *Firmicutes* was considerably affected in P (2 and 4 days) and SJF (8 days) soils at the highest dose (D10). The same trend was observed for the relative abundance of *Planctomycetes* which was lower in D10 P (0 and 30 days) and D1 SJF (2, 4, 8, and 45 days) soils as compared to respective D0. The relative abundance of other phyla fluctuated over time without clear trend in response to leptospermone exposure.

### Impact of leptospermone on bacterial diversity

To further investigate the impact of leptospermone on the microbial community, the bacterial diversity was monitored by 454 pyrosequencing of 16S rRNA amplicons obtained from P and SJF soils exposed (D1 and D10) or not (D0) to leptospermone collected at 4 and 45 days. These points were chosen based on qPCR results (significantly impacted time points). Overall following de-multiplexing and removal of law-quality reads, 454-pyrosequecing generated 189 890 high-quality raw sequence reads of 16S rRNA sequences ranging from 250 and 310 bp in length (Sequence lengths (mean ± std): 266.17 ± 9.17). These sequences were grouped into 4 617 OTUs at 97% nucleotide sequence identity threshold.

In agreement with qPCR results, α-diversity estimated by a range of indices (Table 1) was time-dependent for all soils. In addition, similar diversity was recorded in both soils although they showed different physicochemical properties. For both soils, the Phylogenetic Diversity (PD) Whole Tree, the Chao1 and the Shannon indices gave the same trend indicating that, at 4 days, the bacterial diversity significantly decreased in D1 an D10 microcosms (*p* = 0.002, *p* = 0.002, and *p* = 0.001, respectively). This decrease was accompanied by an increase in the dominance index in both soils (*p* < 0.05). Forty-five days after leptospermone application to the P soil, most of diversity indices estimated from D1 and D10 soils recovered the values measured in D0. In contrast, in SJF soil, for the highest application dose the decrease in the bacterial diversity (PD, Chao1 and Shannon indices, *p* < 0.06) and the increase in the dominance index were persisting. Good's estimator was comprised between 0.76 and 0.89 indicating a relatively good coverage for each sample. Whatever the soil considered, a dose effect was recorded showing that the bacterial diversity was inversely proportional to the dose applied (i.e., D1>D10, PD, Chao1 and Shannon indices, Mann–Whitney U, *p* < 0.05).

**Table 1:** **Richness and diversity indices of the bacterial community were calculated for P and SJF soil microcosms exposed to leptospermone applied at different concentrations (D0, D1, and D10) at 4 and 45 days**.

**Soil and treatment**	**PD whole tree**	**Chao1**	**Dominance**	**Simpson**	**Shannon**	**Good's coverage**
P d4 D0	59.36 ± 1.53^a^	1222 ± 72^a^	0.005 ± 0.001^a^	0.995 ± 0.001^a^	8.59 ± 0.09^a^	0.80 ± 0.009^a^
P d4 D1	40.25 ± 5.91^b^	824 ± 137^b^	0.097 ± 0.070^b^	0.903 ± 0.070^b^	6.27 ± 1.04^b^	0.87 ± 0.021^b^
P d4 D10	38.86 ± 3.92^b^	849 ± 103^b^	0.143 ± 0.062^b^	0.857 ± 0.062^b^	5.55 ± 0.77^c^	0.87 ± 0.017^b^
P d45 D0	71.29 ± 2.44^a^	1457 ± 84^a^	0.003 ± 0.000^a^	0.997 ± 0.000^a^	9.02 ± 0.12^a^	0.76 ± 0.014^a^
P d45 D1	69.86 ± 1.13^a^	1392 ± 64^a^	0.004 ± 0.000^a^	0.996 ± 0.000^b^	8.86 ± 0.06^a^	0.77 ± 0.010^a^
P d45 D10	65.04 ± 3.56^a^	1341 ± 52^a^	0.006 ± 0.001^a^	0.994 ± 0.001^b^	8.57 ± 0.17^b^	0.78 ± 0.012^a^
SJF d4 D0	62.68 ± 0.83^a^	1379 ± 25^a^	0.009 ± 0.002^a^	0.991 ± 0.002^a^	8.48 ± 0.10^a^	0.77 ± 0.001^a^
SJF d4 D1	48.52 ± 0.31^b^	1100 ± 58^b^	0.027 ± 0.006^b^	0.973 ± 0.006^b^	7.18 ± 0.09^b^	0.83 ± 0.006^b^
SJF d4 D10	37.31 ± 2.13^c^	825 ± 54^c^	0.038 ± 0.007^b^	0.962 ± 0.007^c^	6.49 ± 0.21^b^	0.87 ± 0.010^b^
SJF d45 D0	64.19 ± 3.25^a^	1387 ± 26^a^	0.009 ± 0.002^a^	0.991 ± 0.002^a^	8.56 ± 0.11^a^	0.77 ± 0.002^a^
SJF d45 D1	64.48 ± 5.01^a^	1396 ± 14^a^	0.012 ± 0.004^a^	0.988 ± 0.004^b^	8.48 ± 0.22^a^	0.77 ± 0.021^a^
SJF d45 D10	33.29 ± 0.75^b^	679 ± 44^b^	0.022 ± 0.002^b^	0.978 ± 0.002^b^	6.73 ± 0.04^b^	0.89 ± 0.005^b^

*Mean values ± confidence intervals are shown. Similar letters indicate that treatments are not significantly different for each soil and each time point (Kruskal–Wallis test, with Conover's-test for post-hoc multiple comparisons, P < 0.05 were considered as significant)*.

**Figure 2:**
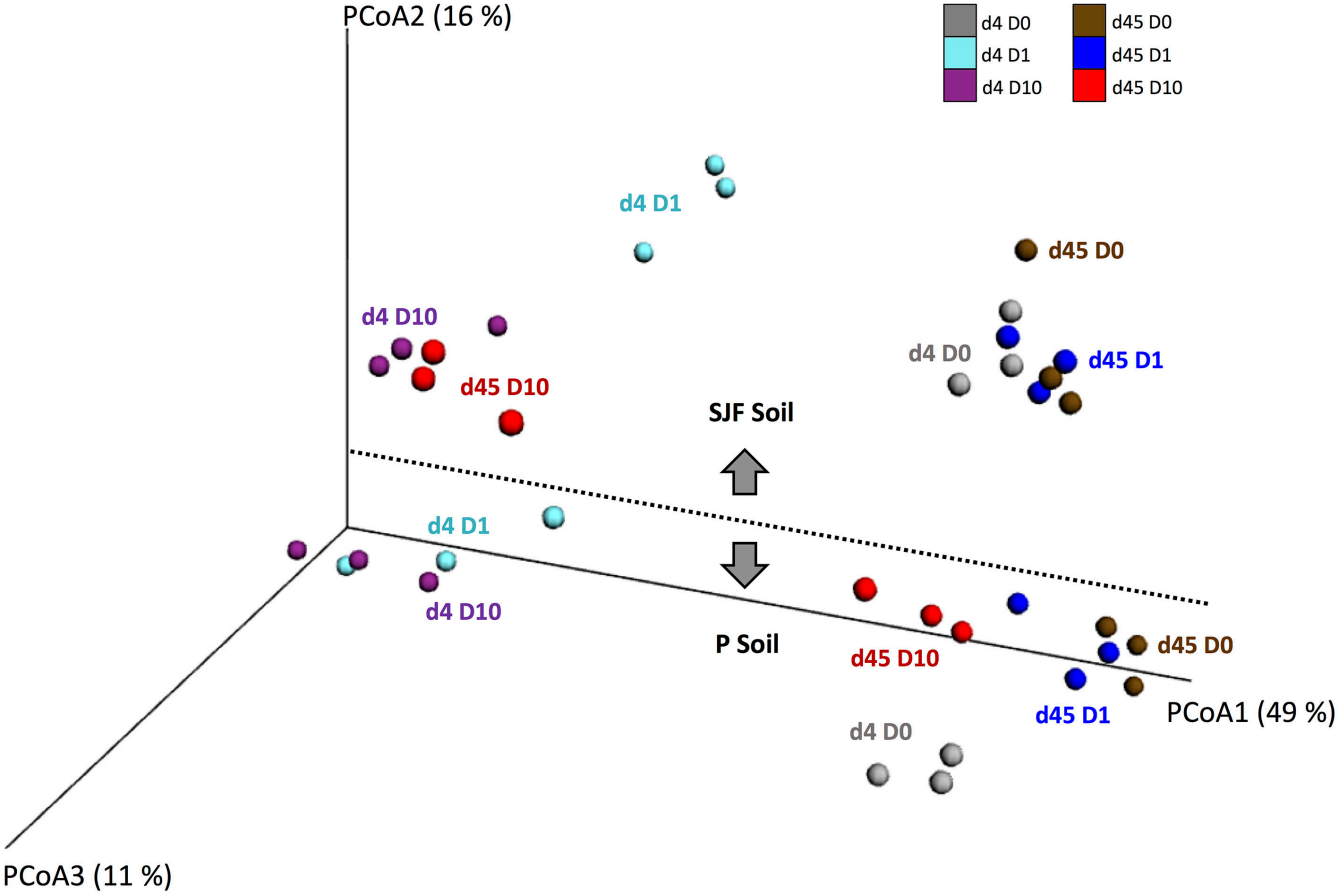
**UniFrac analysis of the effect of leptospermone applied at different concentrations (D0, D1, and D10) on the bacterial community composition of P and SJF soils at 4 and 45 days (d4, d45)**. The first three axes of the PCoA of the weighted UniFrac distance matrix of 16S rRNA amplicon pyrosequencing are shown. The percent of variance explained by each axis is given. For both soils, treatments are as follows: at 4 days for D0, D1, and D10 (gray, cyan, and dark violet), and at 45 days for D0, D1, and D10 (brown, blue and red).

The ecotoxicological impact of leptospermone on the bacterial diversity of P and SJF soils was evaluated using the β-diversity of 16S rRNA barcoding. The analysis of the Principal Coordinates Analyses (PCoA) representing the weighted Unifrac distances showed differences in the bacterial community composition between treatments (D0 vs. D1 and D10), soil type (P vs. SJF) and sampling time (d4 vs. d45) (PERMANOVA, *p* = 0.001) (Figure 2). In addition, a good reproducibility between replicates of each treatment was observed regardless of the soil and the sampling time considered. The bacterial community of the P soil was discriminated from that of SJF along PCoA2 whatever the sampling time and treatment considered. For both soils, 4 days after the treatment, the composition of bacterial community of D1 and D10 soils significantly differed from their respective D0 along PCoA1 accounting for 48% of the variance observed. It is noteworthy that the effect was not dose-dependent (D1 = D10) for P soil whereas a considerable dose effect was observed for SJF soil. Forty-five d after the treatment, the bacterial composition of P soil (D1 and D10) was not significantly different from D0 and was similar to that of D0 at 4 days of incubation. One the contrary, the recovery of the bacterial composition occurred only for the lowest dose of leptospermone applied (D0 = D1) for SJF soil. The bacterial composition of D10 remains different in a similar manner to D1 and D10 after 4 days of exposure (i.e., D10_d45_≠D0_d45_ = D1_d45_).

**Figure 3:**
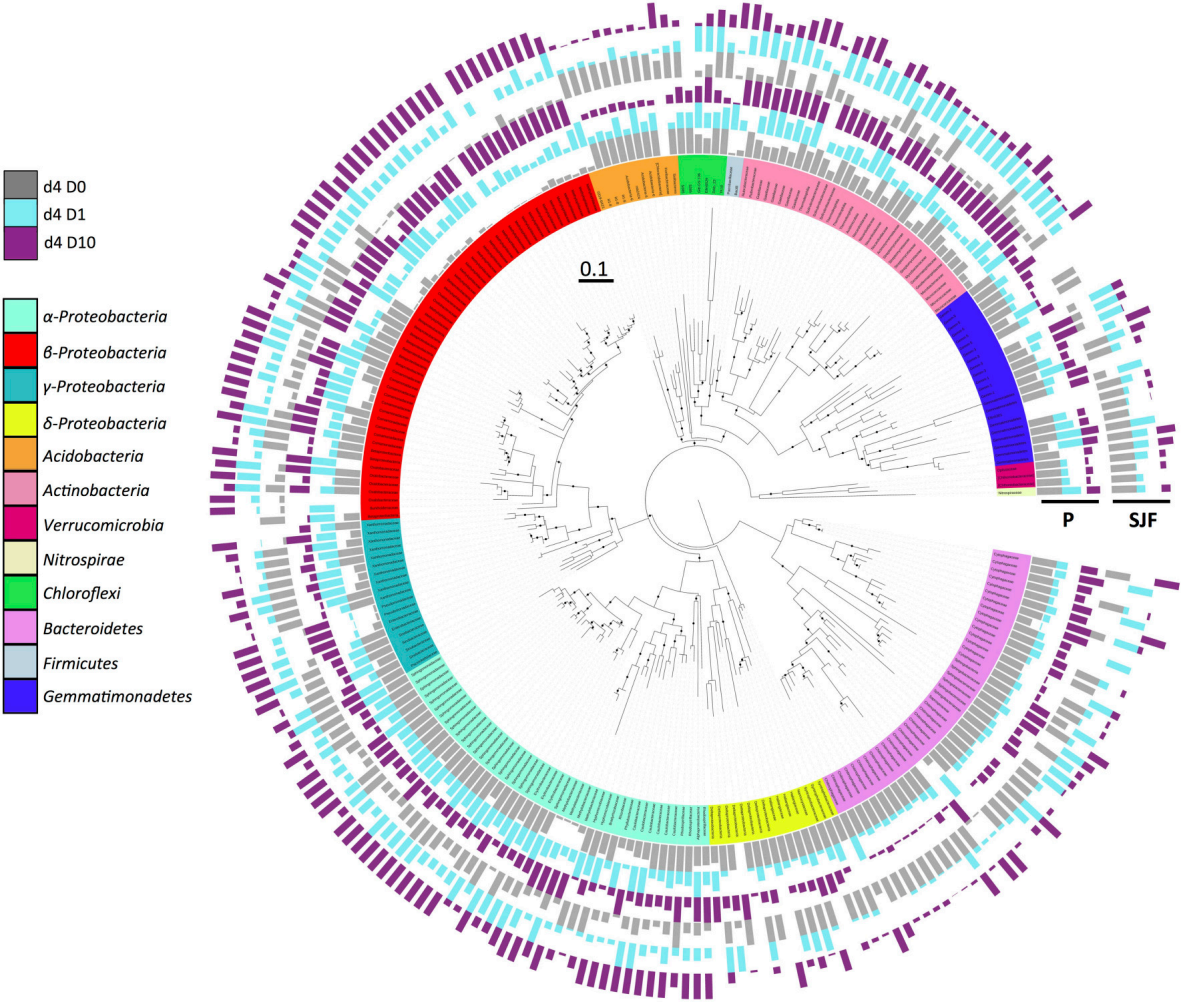
**Phylogenetic relationships and distribution of 16S rRNA OTUs observed in P and SJF soils at 4 days (d4)**. The maximum likelihood phylogeny consists of representative nucleotide sequences for OTUs. Node confidence (*n* = 1000 bootstrap replicates) between 80 and 100% is shown by black dot. The affiliation of the represented OTUs to the main microbial groups (at phylum or class level) is indicated by different colors on the internal ring. Relative abundances of each OTU represented by bar plots are expressed as a proportion of the maximum abundance detected in each treatment, and indicated for P and SJF soils as follows (from inside to outside of the circle): gray, cyan, and dark violet for D0, D1, and D10, respectively.

To further evaluate the ecotoxicological impact of leptospermone application on the composition of the bacterial community, the alignment of the obtained sequences together with known 16S rRNA sequences led to the identification of 33 phyla, 103 classes, 333 families, and 519 of bacterial genera. The phylogenetic tree drawn with iTOL using sequences obtained from P and SJF soils is presented in the Figure 3. The relative abundances of most important 250 OTUs gathered in 12 bacterial phyla (α-, β-, γ-, and δ*-Proteobacteria, Acidobaceteria, Actinobacteria, Verrumicrobiota, Nirospirare, Chhlorflexi, Bacteriodetes, Firmicutes*, and *Gemmatimonadetes*) are shown. The analysis of the phylogenetic tree allowed the discrimination of P and SJF soils before treatment for several phyla (i.e., *Bacteroidetes*). It also revealed that for both soils the composition of bacterial community was deeply affected by the treatment as compared to D0. As an example, for both soils, the relative abundances of an important number of OTUs gathered to β*-Proteobacteria* were higher in D1 and D10 soils than in D0. On the contrary, the relative abundances of almost all OTUs gathered in *Acidobacteria* phylum were decreased in D1 and D10 soils as compared to D0. It is noteworthy that for a number of phyla (including β*-Proteobacteria* and *Acidobacteria*), changes in bacterial composition were proportional to the applied dose of leptospermone (D10>D1).

**Figure 4:**
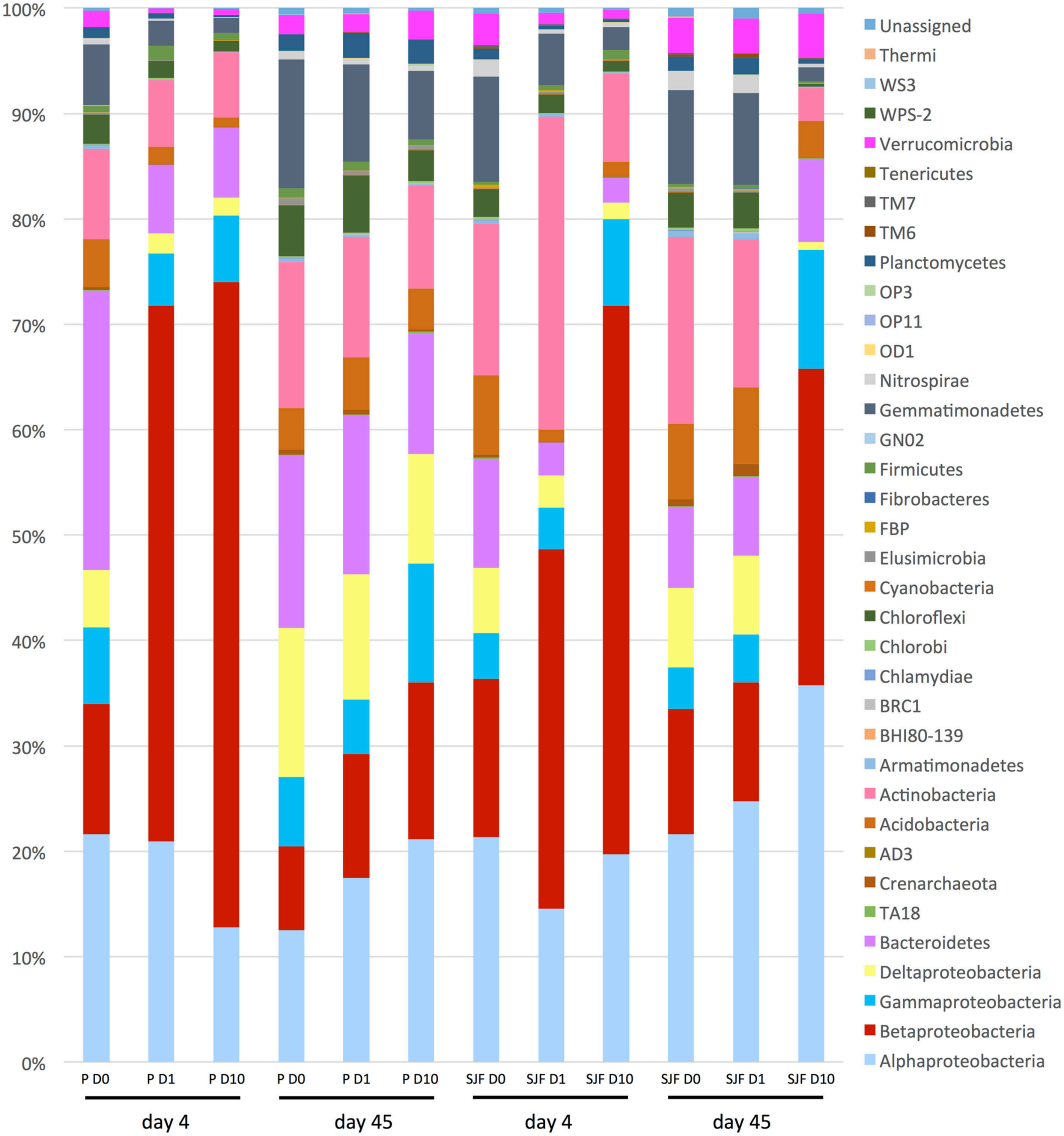
**Relative abundance of bacterial phylum and *Proteobacteria* classes (expressed as % of the total number of OTUs) detected in the P and SJF soils at 4 and 45 days**.

The relative abundances of the 33 phyla identified in P and SJF soils exposed (D1 and D10) or not (D0) to leptospermone for 4 and 45 days are presented in Figure 4. After 4 days of incubation, the bacterial community of both D0 soils was dominated by several bacterial classes including α*-Proteobacteria* (P 21.6%, SJF 21.3%), β*-Proteobacteria* (P 12.4%, SJF 15%), γ*-Proteobacteria* (P 7.3%, SJF 4.35%), *Bacteroidetes* (P 7.58%, SJF 10.32%), *Acidobacteria* (P 7.58%, SJF 7.54%), and *Actinobacteria* (P 4.05%, SJF 8.49%). For both leptospermone-treated soils, the abundance of β*-Proteobacteria* increased by 35 to 60% whereas the abundance of *Acidobacteria* and *Bacteroidetes* decreased by 0.91 to 1.65% and 2.0 to 6.5%, respectively. Although the *Chloroflexi* represented only 3% of the overall bacterial community of the native soils, their abundance was reduced to 1% in the presence of the bioherbicide. Remarkably, at 4 days the abundances of *Opitutales, Gaiellales, Acidimicrobiales*, and *Legionellales* orders were lower in D10 than in D1 while the abundances of *Pseudomonadales* and *Enterobacteriales* orders were higher in D10 than in D1 (Mann–Whitney U, *p* < 0.05). A time-dependent effect was observed in P soil 45 days after treatment. Abundances of bacterial groups (D1 and D10) were close to those of D0 and seemed to recover their initial state. In contrast, the composition of the bacterial community in D10 was different from D0 in SJF soil. Abundances of bacterial groups including *Bacteroidetes* and *Verrumicrobiota* were similar to D0 (45 days), whereas the abundances of α*-Proteobacteria* and β*-Proteobacteria* were higher in D10 than D0 at that time frame. A closer look to α*-Proteobacteria* group revealed an unexpected increase in *Bradyrhizobiaceae* in comparison to D0 (2.95 and 5.93% for D0 and D10, respectively at 45 days). The same trend was observed for *Sphingomonadaceae* (15.63 and 23.65% for D0 and D10, respectively at 45 days).

In order to understand the shifts highlighted by PCoA analysis, the 333 families identified from 454-pyrosequencing were further analyzed with the pamr package. The relative abundances of families like *Opitutaceae, Chitinophagaceae, Bradyrhizobiaceae*, and *Solibacteraceae* decreased after leptospermone treatment (4 days), while others such as *Methylophilaceae, Pseudomonadaceae, Enterobacteriaceae*, and *Xanthomonadaceae* increased at the same time (Supplementary Figure 2). At 45 days, relative abundances of several families (e.g., *Burkholderiaceae* and *Geodermatophilaceae*) almost recovered D0 levels in P soil, but not in SJF soil.

## Discussion

### Fate of leptospermone in soil

As a first step, we monitored the dissipation of leptospermone in the two soils in order to characterize the scenario of exposure of microbial community. Abiotic (i.e., adsorption) and biotic (i.e., biodegradation) processes governing the dissipation of herbicide in soil were investigated. Adsorption isotherms revealed that leptospermone had similar *K*_fa_, 1/n_fa_ (close to 1) and *K*_oc_ indicating a similar and moderate adsorption capacity of leptospermone to P and SJF soils. It is noteworthy that adsorption of leptospermone observed in P soil (*K*_oc_ = 143.86 mL g^−1^) is similar to that of sulcotrione (*K*_oc_ = 144 mL g^−1^), a synthetic β-triketone herbicide, reported in an earlier study (Chaabane et al., 2005). It is not surprising to have similar adsorption behavior of leptospermone in both soils because they have almost identical soil pH (i.e., P pH 7.62 vs. SJF pH 8.1) and organic carbon content (i.e., P 0.98% vs. SJF 0.9%) which are two parameters known to influence adsorption of herbicides on soil (Dyson et al., 2002). Despite the moderate affinity of leptospermone for P and SJF soils, the bioherbicide dissipated rapidly from both soils with half-lives (DT_50_) ranging from 4 to 10 days depending on the soil (i.e., soil effect: P-DT_50_ < SJF-DT_50_) and on the treatment (i.e., dose effect: D1-DT_50_ < D10-DT_50_). Leptospermone persisted longer in SJF than in P soil, particularly in D10 SJF soil where approximately 10 μg g^−1^ of leptospermone remained throughout the experiment. Since, the level of leptospermone did not decrease in sterilized soil microcosms its dissipation is dependent on biotic processes. This is consistent with previous reports on the biotic degradation of synthetic β-triketone herbicides (sulcotrione and mesotrione) in soil (Batisson et al., 2009; Crouzet et al., 2010; Calvayrac et al., 2012). Leptospermone DT_50_ was in the same range as sulcotrione and mesotrione, ranging from 8 to 65 days and 6 to 34 days, respectively (Dyson et al., 2002; Chaabane et al., 2008; Calvayrac et al., 2012). Based on the dissipation kinetics, we conclude that the scenario of exposure of soil microorganisms to this natural β-triketone was differing between the two soils (P < SJF).

### Estimation of the ecotoxicological impact of leptospermone

The impact of leptospermone on the composition and on the diversity of the soil microbial community was assessed by qPCR targeting 11 microbial groups and 454 pyrosequencing, respectively. *Actinobacteria* and *Bacteroidetes* co-dominated the total bacterial community in both P and SJF soils as described in a range of other soils (Janssen, 2006; Acosta-Martinez et al., 2008; Wessen et al., 2010; Petric et al., 2011; Merlin et al., 2015). 16S rRNA metabarcoding confirmed that these two groups as well as *Proteobacteria* and *Acidobacteria* phyla were dominating the bacterial community of both soils. This divergence between qPCR and 454 pyrosequencing could result from the degeneracy of the primers used to quantify the eleven microbial groups causing bias in the quantification as shown earlier by Bru et al. (2008). The abundances of several groups (β- and γ-*Proteobacteria*) increased in both soils, whereas the abundances of several others (*Acidobacteria, Firmicutes*, and *Planctomyectes*) decreased in leptospermone-treated soils. There was a transient loss in α-diversity for both soils. Furthermore, β-diversity results confirmed significant changes in the composition of the bacterial community in both P and SJF soils at 4 days, which were recovered after 45 days of incubation in P (D1 and D10) and SJF (D1) soils, but not in D10 SJF soil. Altogether, leptospermone significantly reduced the bacterial diversity and altered the soil bacterial composition when less than 50% of the bioherbicide is dissipated. On the other hand, the bacterial diversity and composition of P and SJF soils recovered when leptospermone dissipated entirely from the P microcosm. However, this recovery was not achieved in the SJF soil treated with D10, where 10 μg g^−1^ of leptospermone was still remained by the end of the experiment.

Although soil microorganisms are considered as NTO of β-triketone herbicides, most of them, express the *hppd* gene, coding for the HPPD enzyme targeted by these herbicides (Milcamps and de Bruijn, 1999; Moran, 2005). The observed loss in soil microbial diversity might be due to a disruption of bacterial tyrosine catabolism following the inhibition of HPPD by leptospermone. This may diminish the growth rate of sensitive populations (Milcamps and de Bruijn, 1999; Moran, 2005; Yang et al., 2007) while allowing leptospermone-tolerant microorganisms to expend. Changes in microbial diversity can also be caused by indirect toxic effect of leptospermone favoring the growth of saprophytic bacterial species feeding on dead leptospermone-sensitive microorganisms. The combination of direct and indirect ecotoxicological effects may explain changes caused by leptospermone on soil microbial diversity. While leptospermone caused a transient alteration of the composition and diversity of the soil microbial community, the biological significance of the ecotoxicological impact remains difficult to interpret in the absence of (i) normal operating range defining acceptable values for the measured parameters and (ii) an assessment of functional consequences of the changes observed.

The strong biotic control over the dissipation of leptospermone suggests that β-triketone-degrading populations can emerge in soils exposed to this chemistry. This hypothesis was tested by identifying bacterial groups with the most positive response in the presence of leptospermone. Within β- and γ*-Proteobacteria*, the abundance of *Methylophilales, Burkholderiales, Pseudomonadales*, and *Enterobacteriales* increased significantly to leptospermone exposure. Relative groups belonging to these two orders are stress tolerant and degrade several contaminants including polycyclic aromatic hydrocarbons and various pesticides (Zhang et al., 2011, 2012; Calvayrac et al., 2012; Giri et al., 2013; Li et al., 2014; Fang et al., 2015). Since microbial degradation of pesticides is an adaptive response to cope with the stress imposed by xenobiotics (Crouzet et al., 2010; Olchanheski et al., 2014; Bardot et al., 2015), the emergence of degraders in the microbial community is a biomarker of exposure. Such responses in microbial community structure have already been described for other herbicides including mesotrione, glyphosate, s-triazine, and sulfonylurea (Busse et al., 2001; Ros et al., 2006; Weaver et al., 2007; Crouzet et al., 2010). However, the abundance of degrading populations remains rather low (i.e., between 10^3^ and 10^4^ cells per g of soil) even in soil conditioned for rapid pesticide biodegradation (Piutti et al., 2003). Often emergence of degraders in microbial communities cannot be detected using tools targeting the overall microbial community because they might be hidden by dominant populations as demonstrated for chlordecone (Merlin et al., 2015). Therefore, the emergence of leptospermone degraders may make a negligible contribution to the overall microbial diversity of a soil. Therefore, the microbial biodegradation of leptospermone needs to be further studied using traditional enrichment culture techniques to better understand the environmental profile of leptospermone.

## Conclusion

To our best knowledge this is the first study investigating the fate and the ecotoxicological impact of leptospermone on the composition and diversity of the soil microbial community. Leptospermone altered composition and diversity of soil microbial community despite of its relatively rapid rate of degradation. These changes were transient and when leptospermone was fully dissipated, the microbial community recovered its initial composition and diversity. However, the recovery cannot be reached in soil microcosms where leptospermone remained. Further work need to be done to test the ecotoxicological impact of natural β-triketone on a wider range of soil classes. In addition, it would be interesting to contrast the ecotoxicological impact of synthetic (sulcotrione and mesotrione) and natural β-triketones on soil microbial community.

## Author contributions

SR, MD, LB, CC, CB, JC, FD, FM: Substantial contributions to the conception or design of the work; or the acquisition, analysis, or interpretation of data for the work; and drafting the work or revising it critically for important intellectual content; and final approval of the version to be published; and agreement to be accountable for all aspects of the work in ensuring that questions related to the accuracy or integrity of any part of the work are appropriately investigated and resolved.

### Conflict of interest statement

The authors declare that the research was conducted in the absence of any commercial or financial relationships that could be construed as a potential conflict of interest.
