# Supplementary material for: Ecotoxicological Impact of the Bioherbicide Leptospermone on the Microbial Community of Two Arable Soils
Source: Front Microbiol. 2016 May 24;7:775. doi: 10.3389/fmicb.2016.00775 (PMC4877392; doi:10.3389/fmicb.2016.00775)
Supplement: Supplementary file 1 [file Presentation1.PPTX]

## Slide 1
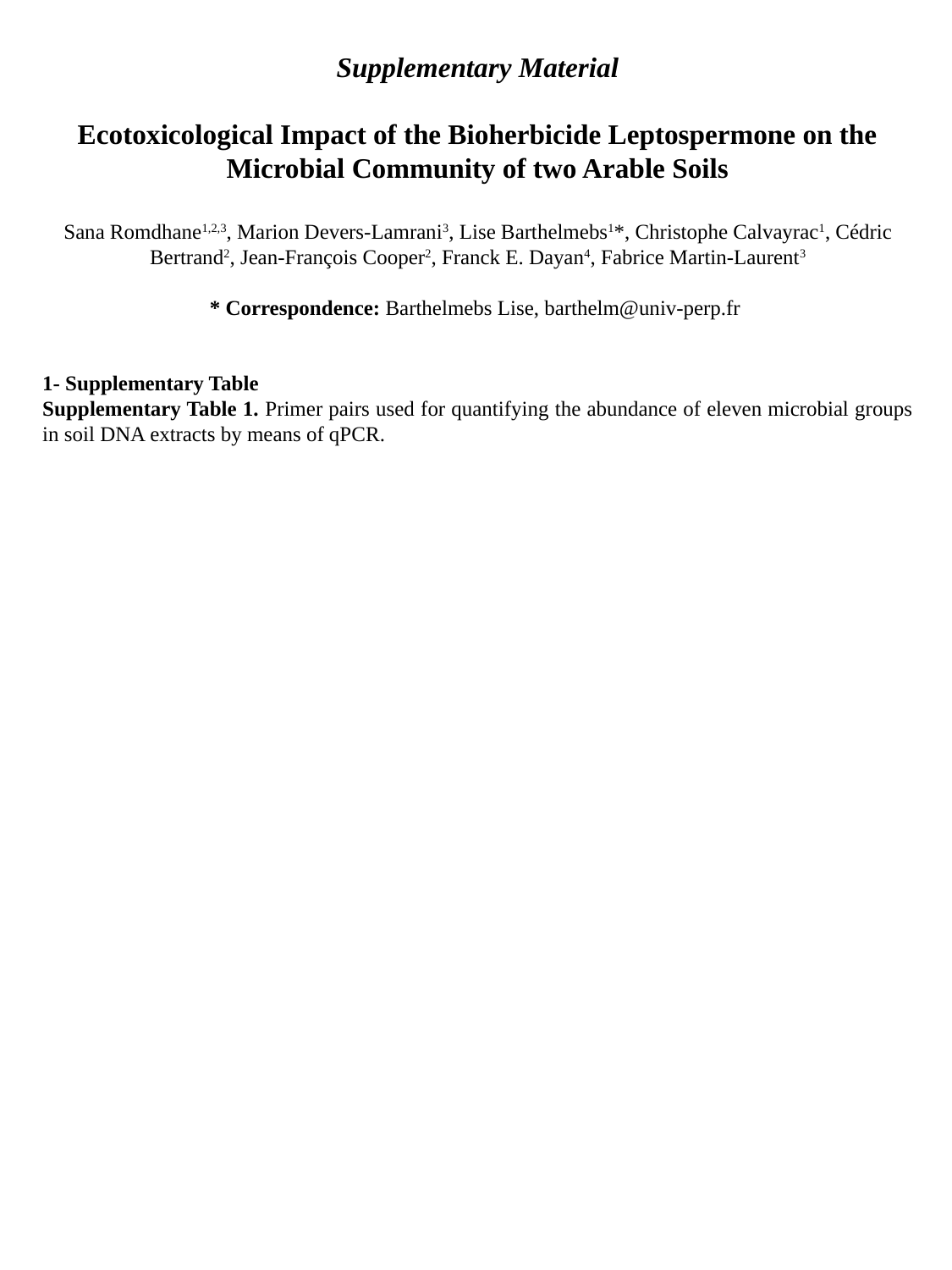

Supplementary Material
Ecotoxicological Impact of the Bioherbicide Leptospermone on the Microbial Community of two Arable Soils
Sana Romdhane1,2,3, Marion Devers-Lamrani3, Lise Barthelmebs1*, Christophe Calvayrac1, Cédric Bertrand2, Jean-François Cooper2, Franck E. Dayan4, Fabrice Martin-Laurent3
* Correspondence: Barthelmebs Lise, barthelm@univ-perp.fr
1- Supplementary Table
Supplementary Table 1. Primer pairs used for quantifying the abundance of eleven microbial groups in soil DNA extracts by means of qPCR.

## Slide 2
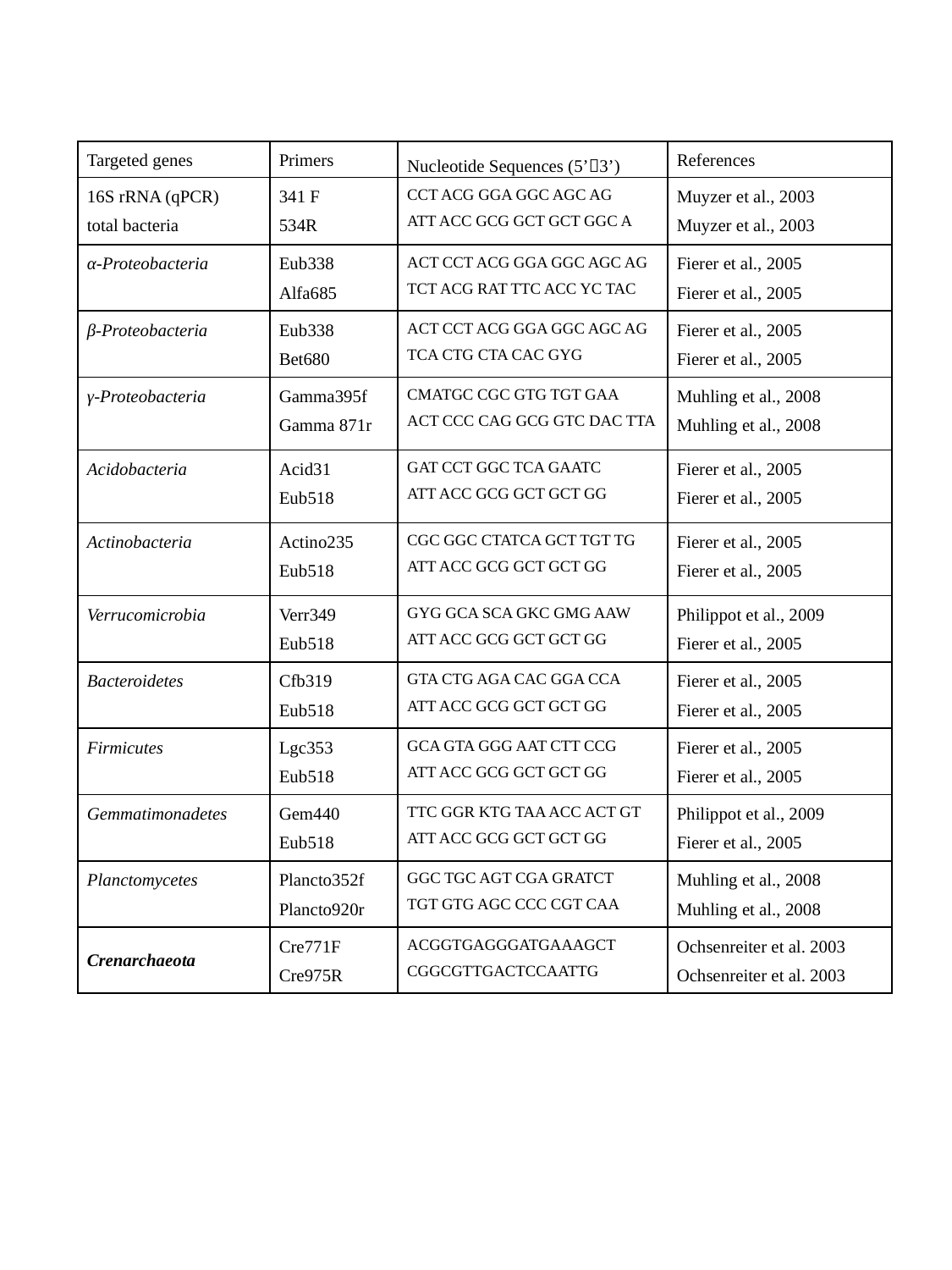

| Targeted genes | Primers | Nucleotide Sequences (5’3’) | References |
| --- | --- | --- | --- |
| 16S rRNA (qPCR) total bacteria | 341 F 534R | CCT ACG GGA GGC AGC AG ATT ACC GCG GCT GCT GGC A | Muyzer et al., 2003 Muyzer et al., 2003 |
| α-Proteobacteria | Eub338 Alfa685 | ACT CCT ACG GGA GGC AGC AG TCT ACG RAT TTC ACC YC TAC | Fierer et al., 2005 Fierer et al., 2005 |
| β-Proteobacteria | Eub338 Bet680 | ACT CCT ACG GGA GGC AGC AG TCA CTG CTA CAC GYG | Fierer et al., 2005 Fierer et al., 2005 |
| γ-Proteobacteria | Gamma395f Gamma 871r | CMATGC CGC GTG TGT GAA ACT CCC CAG GCG GTC DAC TTA | Muhling et al., 2008 Muhling et al., 2008 |
| Acidobacteria | Acid31 Eub518 | GAT CCT GGC TCA GAATC ATT ACC GCG GCT GCT GG | Fierer et al., 2005 Fierer et al., 2005 |
| Actinobacteria | Actino235 Eub518 | CGC GGC CTATCA GCT TGT TG ATT ACC GCG GCT GCT GG | Fierer et al., 2005 Fierer et al., 2005 |
| Verrucomicrobia | Verr349 Eub518 | GYG GCA SCA GKC GMG AAW ATT ACC GCG GCT GCT GG | Philippot et al., 2009 Fierer et al., 2005 |
| Bacteroidetes | Cfb319 Eub518 | GTA CTG AGA CAC GGA CCA ATT ACC GCG GCT GCT GG | Fierer et al., 2005 Fierer et al., 2005 |
| Firmicutes | Lgc353 Eub518 | GCA GTA GGG AAT CTT CCG ATT ACC GCG GCT GCT GG | Fierer et al., 2005 Fierer et al., 2005 |
| Gemmatimonadetes | Gem440 Eub518 | TTC GGR KTG TAA ACC ACT GT ATT ACC GCG GCT GCT GG | Philippot et al., 2009 Fierer et al., 2005 |
| Planctomycetes | Plancto352f Plancto920r | GGC TGC AGT CGA GRATCT TGT GTG AGC CCC CGT CAA | Muhling et al., 2008 Muhling et al., 2008 |
| Crenarchaeota | Cre771F Cre975R | ACGGTGAGGGATGAAAGCT CGGCGTTGACTCCAATTG | Ochsenreiter et al. 2003 Ochsenreiter et al. 2003 |

## Slide 3
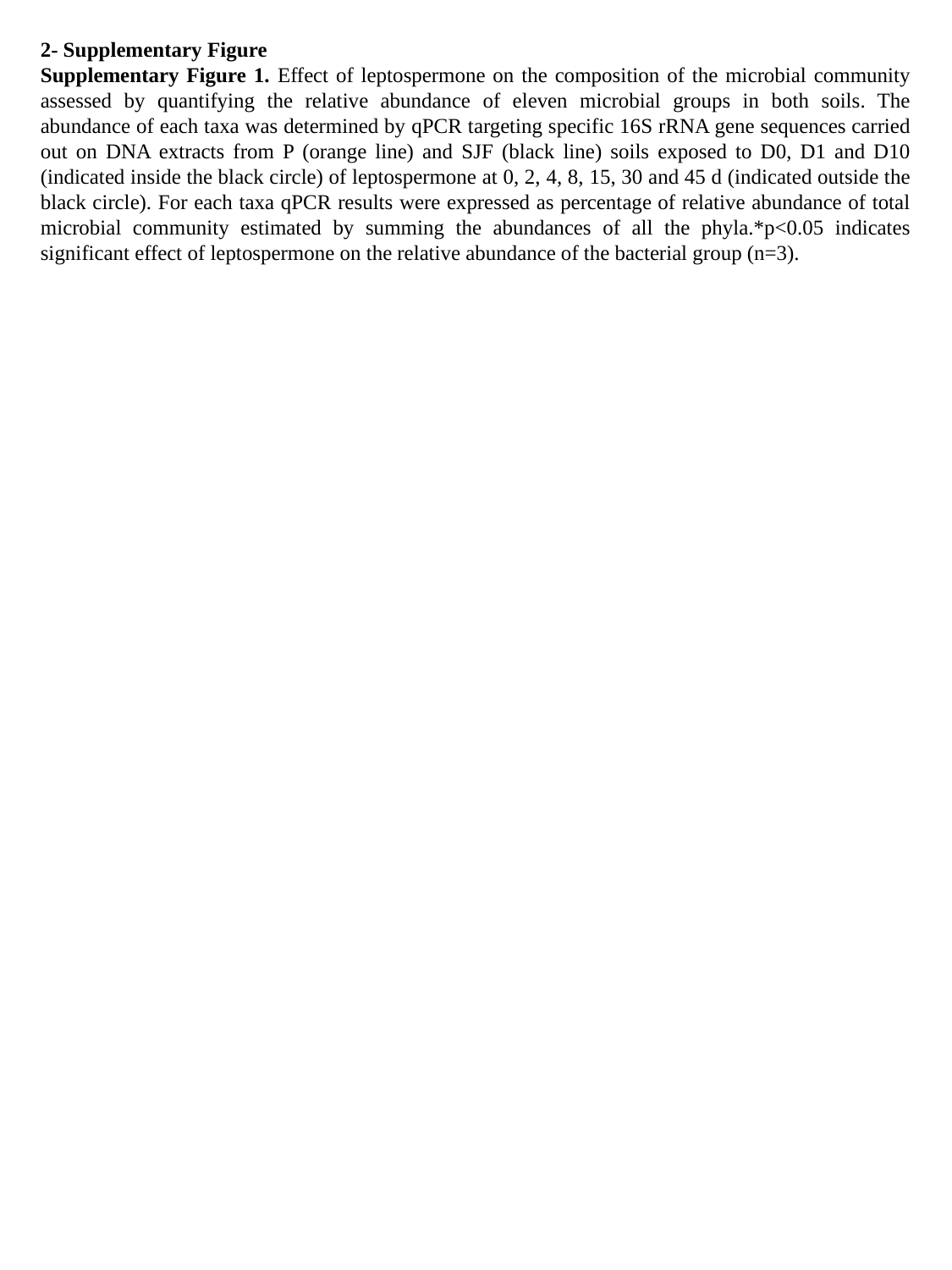

2- Supplementary Figure
Supplementary Figure 1. Effect of leptospermone on the composition of the microbial community assessed by quantifying the relative abundance of eleven microbial groups in both soils. The abundance of each taxa was determined by qPCR targeting specific 16S rRNA gene sequences carried out on DNA extracts from P (orange line) and SJF (black line) soils exposed to D0, D1 and D10 (indicated inside the black circle) of leptospermone at 0, 2, 4, 8, 15, 30 and 45 d (indicated outside the black circle). For each taxa qPCR results were expressed as percentage of relative abundance of total microbial community estimated by summing the abundances of all the phyla.*p<0.05 indicates significant effect of leptospermone on the relative abundance of the bacterial group (n=3).

## Slide 4
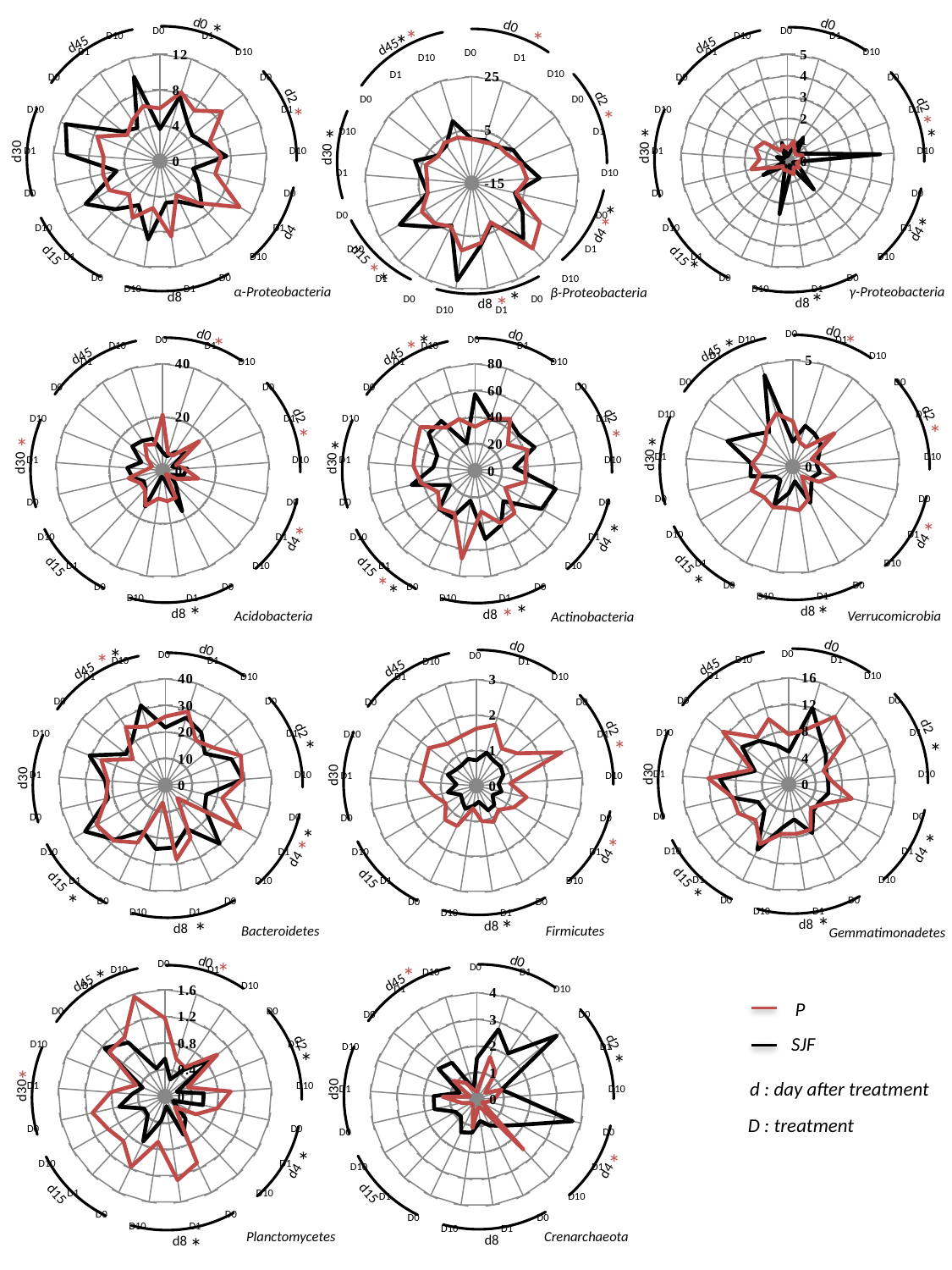

### Chart
| Category | SJF α-proteobacteria | Sp α-proteobacteria |
|---|---|---|
| D0 | 3.597947555734619 | 5.925099800120304 |
| D1 | 7.62649913209847 | 8.0974099409542 |
| D10 | 5.425429315807193 | 6.868073123600496 |
| D0 | 4.65474333553058 | 8.945875457607853 |
| D1 | 5.514069416022096 | 6.033710077126446 |
| D10 | 7.484037992374836 | 6.9028852886939225 |
| D0 | 3.832175040047447 | 6.38541814624307 |
| D1 | 5.0284095978895476 | 10.354261157102352 |
| D10 | 6.946590120620911 | 6.456635926307908 |
| D0 | 5.056178724390501 | 4.249043355803001 |
| D1 | 4.717909450954236 | 8.573301156043286 |
| D10 | 8.915385449621635 | 5.346129041592474 |
| D0 | 5.505660275653908 | 7.0700926262513715 |
| D1 | 7.380065023867353 | 5.099141634026555 |
| D10 | 9.641004063016148 | 6.556929309979608 |
| D0 | 4.993830743148179 | 6.57998456687098 |
| D1 | 10.47583580119134 | 6.3540492930101395 |
| D10 | 11.379148625636066 | 7.56669328572212 |
| D0 | 5.311796023040897 | 4.715344976197703 |
| D1 | 4.5111704387590565 | 5.513777375256338 |
| D10 | 9.92101053094583 | 6.471747112386744 |
### Chart
| Category | SJF γ-proteobacteria | Sp γ-proteobacteria |
|---|---|---|
| D0 | 0.2242550396074184 | 0.5902846864772182 |
| D1 | 0.4887467101798719 | 0.9770861977815005 |
| D10 | 1.290562369830404 | 0.5747451034972676 |
| D0 | 0.5602153711996466 | 0.4912894317238938 |
| D1 | 0.9501289395324298 | 0.7606008063253086 |
| D10 | 4.345482835789499 | 0.6316903488951506 |
| D0 | 0.2586339988702539 | 0.4391880387486077 |
| D1 | 0.6847144411406342 | 0.6289084727773112 |
| D10 | 1.8058617882699965 | 0.41952020414016544 |
| D0 | 0.20284301431665697 | 0.6785373548938016 |
| D1 | 0.6495889372663582 | 0.5037030723985007 |
| D10 | 2.501771330746668 | 0.4362029342124324 |
| D0 | 0.22960538698365088 | 0.44103671976444075 |
| D1 | 0.3932884003959402 | 0.32691585983130184 |
| D10 | 1.3057174156631588 | 0.5782086245067779 |
| D0 | 0.1875767906108359 | 1.738129021646072 |
| D1 | 0.25919322804117795 | 1.3051552507878494 |
| D10 | 0.542760046367059 | 1.6182527110322462 |
| D0 | 0.1994731060301402 | 1.4115577764240579 |
| D1 | 0.21903887938116956 | 0.5976265986432423 |
| D10 | 0.8841783128088335 | 0.7723538003101463 |d0
*
d0
*
*
*
d45
d45
d2
*
d2
*
*
*
d30
d30
*
*
d4
d4
d15
d15
*
*
α-Proteobacteria
β-Proteobacteria
d8
d8
d0
d45
d2
d30
d4
d15
d8
*
*
*
*
γ-Proteobacteria
*
### Chart
| Category | SJF β-proteobacteria | Sp β-proteobacteria |
|---|---|---|
| D0 | 1.4987091468809213 | 1.46726898942183 |
| D1 | 1.835214562094895 | 1.0481730289467368 |
| D10 | 1.5768194165530531 | 2.220818667801454 |
| D0 | 4.853492690828154 | 2.1118851440040967 |
| D1 | 5.629981534606676 | 4.794022794697182 |
| D10 | 10.520476854836422 | 5.790078122534115 |
| D0 | 1.5388940021199367 | 1.9849835842888452 |
| D1 | 6.988898511070389 | 14.475584927709216 |
| D10 | 13.383593236397358 | 18.52579459463204 |
| D0 | 1.8818089012087535 | 1.2976520958899944 |
| D1 | 8.402402615135012 | 7.763465744671033 |
| D10 | 22.015579714269826 | 10.622336309927698 |
| D0 | 2.6939709296069254 | 3.0981300024543303 |
| D1 | 7.453516977173792 | 5.6706385479856545 |
| D10 | 16.21376772599289 | 6.63778086958974 |
| D0 | 1.9443397086207137 | 2.0088082848150575 |
| D1 | 3.695277937956847 | 1.8090421367976641 |
| D10 | 7.8339077399889545 | 3.5285025952947007 |
| D0 | 1.516228600125732 | 1.12192363567728 |
| D1 | 1.9833734960046419 | 3.269884352837243 |
| D10 | 9.343868825451183 | 2.87497738494233 |*
*
### Chart
| Category | SJF Verrucomicrobia | Sp Verrucomicrobia |
|---|---|---|
| D0 | 1.1672116474276955 | 2.1247878748628195 |
| D1 | 1.999652042798923 | 1.176223025735465 |
| D10 | 1.8425242226381238 | 1.10087114584602 |
| D0 | 1.6819764927577188 | 2.5159371216630237 |
| D1 | 1.3087637318449052 | 0.9504273027675635 |
| D10 | 1.1299964635674118 | 1.3733944424540672 |
| D0 | 1.304795289928798 | 2.0344558910612327 |
| D1 | 1.0353039650492262 | 1.4274938040754537 |
| D10 | 1.282493760990206 | 0.6224845711523216 |
| D0 | 1.888365237832872 | 1.700061374934215 |
| D1 | 0.6967343590661776 | 2.0705737168903613 |
| D10 | 1.2636713212233937 | 1.950001681128374 |
| D0 | 1.9998559955152733 | 2.1181645559152553 |
| D1 | 0.8455679315810931 | 1.9626001316347612 |
| D10 | 0.9450209677723607 | 2.2247071973397152 |
| D0 | 2.0303846474892526 | 1.5776314158209914 |
| D1 | 1.9529778197357397 | 1.902605085694331 |
| D10 | 3.283931603527141 | 1.6402936547993698 |
| D0 | 2.3892165095108124 | 1.671302699710816 |
| D1 | 1.9616241178085598 | 2.145213132962288 |
| D10 | 4.483302833500166 | 2.601982755140039 |d0
d45
d2
d30
d4
d15
d8
*
*
*
*
*
*
*
Verrucomicrobia
### Chart
| Category | SJF Actinobacteria | Sp Actinobacteria |
|---|---|---|
| D0 | 57.19356297291309 | 32.763463628198785 |
| D1 | 39.92264512116192 | 40.63907594929788 |
| D10 | 45.6644675791806 | 46.76121382863067 |
| D0 | 42.32899209656281 | 31.487096278687424 |
| D1 | 47.94096859556426 | 42.33854805431264 |
| D10 | 29.40734196740809 | 37.72452497147015 |
| D0 | 62.11880867477996 | 38.786509422786814 |
| D1 | 57.604160349396075 | 26.235983942212467 |
| D10 | 31.34723247803587 | 43.65804531103487 |
| D0 | 45.31522233169812 | 43.93810986750926 |
| D1 | 51.93280871274254 | 31.09855973798307 |
| D10 | 22.88411830102872 | 66.83129692307774 |
| D0 | 38.870664642566 | 35.55659555350234 |
| D1 | 39.42988840519842 | 38.78655201512616 |
| D10 | 21.816184780121784 | 32.107596669382474 |
| D0 | 48.77811657168157 | 41.60793482648521 |
| D1 | 31.406640279302895 | 46.23926350813562 |
| D10 | 30.385475335977585 | 47.729043650912004 |
| D0 | 44.549913969849676 | 52.303113709269425 |
| D1 | 44.936246692511354 | 38.902431113870456 |
| D10 | 21.25204375328445 | 40.38394449167628 |d0
d45
d2
d30
d4
d15
d8
*
*
*
*
*
*
*
*
*
Actinobacteria
d0
d45
d2
d30
d4
d15
d8
*
*
*
*
*
Acidobacteria
### Chart
| Category | SJF Acidobacteria | Sp Acidobacteria |
|---|---|---|
| D0 | 6.903486794915581 | 20.95625255333892 |
| D1 | 5.546057082577597 | 6.783259477416787 |
| D10 | 8.3233573333226 | 6.768121461620193 |
| D0 | 10.71271795908155 | 17.618224079178066 |
| D1 | 3.8783685604948652 | 5.414355564316847 |
| D10 | 9.26241331153885 | 8.338099316917 |
| D0 | 7.9970526386497145 | 13.687090597704694 |
| D1 | 3.64079026320597 | 6.3500104485525295 |
| D10 | 7.25426019694644 | 1.8093010758241275 |
| D0 | 16.870392648768036 | 11.326356743283563 |
| D1 | 3.1982469877691413 | 11.602392225812375 |
| D10 | 2.404710758757405 | 10.616366265336568 |
| D0 | 15.046232016929006 | 14.605914842408072 |
| D1 | 9.389447960529626 | 9.795154413857434 |
| D10 | 8.129366542491217 | 9.544961873250129 |
| D0 | 11.56851450495117 | 13.111963219024489 |
| D1 | 13.24478964829627 | 5.772063907131454 |
| D10 | 8.576539772406232 | 4.239065345922173 |
| D0 | 14.522210967158577 | 7.4842708716294695 |
| D1 | 13.471675537801536 | 11.625605400773049 |
| D10 | 12.48805726976094 | 10.248463159895298 |
### Chart
| Category | SJF Gemmatimonadetes | Sp Gemmatimonadetes |
|---|---|---|
| D0 | 4.915683127671859 | 7.484543811449147 |
| D1 | 12.02792361508491 | 8.612782562364286 |
| D10 | 8.482425470969153 | 12.297742618502516 |
| D0 | 7.1208731967362455 | 10.712282125100568 |
| D1 | 6.00711492934246 | 5.599537413268852 |
| D10 | 5.9465006182065805 | 6.4939048584039485 |
| D0 | 6.097940157224246 | 9.705625963481564 |
| D1 | 5.307910704325727 | 5.928931522469044 |
| D10 | 5.416701620732492 | 4.858015891371985 |
| D0 | 8.118424728646469 | 7.5655895453961675 |
| D1 | 5.328342460798004 | 7.534060177253051 |
| D10 | 6.622171375684861 | 7.551076392974043 |
| D0 | 10.92178703769542 | 9.974469164508545 |
| D1 | 5.397916204194244 | 7.349958559165813 |
| D10 | 5.347536410390177 | 8.757922214722196 |
| D0 | 8.860891427826376 | 8.694410090549546 |
| D1 | 10.39506577800282 | 12.157116144718868 |
| D10 | 5.488784887464909 | 5.989918091421308 |
| D0 | 8.99029109797992 | 12.697470304557523 |
| D1 | 7.906674815470002 | 8.53968933248471 |
| D10 | 6.144808290385009 | 10.25466881715227 |*
*
*
Gemmatimonadetes
d0
d45
d2
d30
d4
d15
d8
*
### Chart
| Category | SJF Bacteroidetes | Sp Bacteroidetes |
|---|---|---|
| D0 | 21.67775144122247 | 25.808106562194567 |
| D1 | 26.57040166574212 | 28.970295523069133 |
| D10 | 23.974104753634048 | 20.381419838733304 |
| D0 | 19.070993776994914 | 22.763712738253574 |
| D1 | 26.77283586432423 | 30.511383798351844 |
| D10 | 29.179560016266585 | 29.000044147389726 |
| D0 | 15.802034198653102 | 21.89783336228929 |
| D1 | 17.1405585147235 | 32.56262820266304 |
| D10 | 30.019930378889928 | 6.58425745496965 |
| D0 | 18.17952270793238 | 21.89783336228929 |
| D1 | 23.647547131784354 | 28.266100813566933 |
| D10 | 24.2237379958203 | 6.58425745496965 |
| D0 | 19.365608363010328 | 23.955259512306334 |
| D1 | 27.87600841823245 | 28.43618429598999 |
| D10 | 34.800918322854194 | 29.782831420595052 |
| D0 | 22.04916497179931 | 22.854626832997443 |
| D1 | 22.646761379074718 | 21.636042326927292 |
| D10 | 30.595238904584132 | 25.846295717745267 |
| D0 | 18.79516735957909 | 15.672144177354875 |
| D1 | 21.65545934676229 | 26.415895938809665 |
| D10 | 31.394590687267762 | 23.00597048680359 |
### Chart
| Category | SJF Firmicutes | Sp Firmicutes |
|---|---|---|
| D0 | 0.7196683696033127 | 1.6141894779296884 |
| D1 | 0.9778346683550326 | 1.79908337135755 |
| D10 | 0.8587176354283674 | 1.2741166438691658 |
| D0 | 0.8757513259445205 | 1.4699111566660032 |
| D1 | 0.819025744545274 | 2.581681102627369 |
| D10 | 0.6271198410952806 | 0.957070051662891 |
| D0 | 0.7281878993412675 | 1.4519470023087202 |
| D1 | 0.5227481198038878 | 1.2179319708082998 |
| D10 | 0.7408457996063768 | 0.918746210592142 |
| D0 | 0.762893154311589 | 1.124041872063984 |
| D1 | 0.45494919927027133 | 1.0040129043932329 |
| D10 | 0.5703859509935126 | 0.6380203741220781 |
| D0 | 0.7193795061951048 | 1.2560747178099427 |
| D1 | 0.578674708519284 | 1.3016088741437717 |
| D10 | 0.504616999426268 | 0.9928700432210715 |
| D0 | 0.8235841413691195 | 1.2298443363295364 |
| D1 | 0.5621636865744246 | 1.5778572711036933 |
| D10 | 0.8632289483870117 | 1.5664521260615212 |
| D0 | 0.73074780090405 | 1.7091292887348404 |
| D1 | 0.713438986400058 | 1.4406502610131706 |
| D10 | 0.7966870338856347 | 1.4611201941641145 |d0
d45
d2
d30
d4
d15
d8
*
*
Firmicutes
*
*
*
*
*
*
*
d0
d45
d2
d30
d4
d15
d8
*
Bacteroidetes
### Chart
| Category | SJF Planctomycètes | Sp Planctomycètes |
|---|---|---|
| D0 | 0.5642754298286886 | 1.17003088815533 |
| D1 | 0.2631880415301764 | 0.5832523766267759 |
| D10 | 0.47420813435530906 | 0.5258458595633396 |
| D0 | 0.8959831855733963 | 1.0038383512781808 |
| D1 | 0.18888392096439038 | 0.36584911635080297 |
| D10 | 0.584059381011977 | 0.9880204881001019 |
| D0 | 0.584258641945851 | 0.8059162175909002 |
| D1 | 0.13666795165373788 | 0.5373882059951336 |
| D10 | 0.4514119679719217 | 0.2169046736067312 |
| D0 | 0.6384282094842206 | 1.1071521925315593 |
| D1 | 0.15000699520572824 | 1.269257490728447 |
| D10 | 0.36715251651012165 | 0.6913613273731176 |
| D0 | 0.7530023610470296 | 1.1751295671426274 |
| D1 | 0.3800308255950333 | 0.9159743992017877 |
| D10 | 0.36316864322915504 | 0.9823535012650667 |
| D0 | 0.7043748736682002 | 1.1127357856474838 |
| D1 | 0.49103709440819215 | 0.8191113356462525 |
| D10 | 0.36064817839909213 | 0.46646720712905115 |
| D0 | 1.1598306125433877 | 1.0700764040800663 |
| D1 | 0.9768104575450148 | 1.0744392407099281 |
| D10 | 0.4354589380783644 | 1.563075838251964 |
### Chart
| Category | SJF Achaea | Sp Archaea |
|---|---|---|
| D0 | 1.537448474194406 | 0.28841245147839883 |
| D1 | 2.7418373583761344 | 1.6615362714538573 |
| D10 | 2.087383768281088 | 1.2235681873260038 |
| D0 | 3.8294250025934833 | 0.39415134977964994 |
| D1 | 0.9898587627583485 | 0.9909645720402186 |
| D10 | 1.5130107179045018 | 0.6938492907280057 |
| D0 | 3.6802953513384944 | 0.4166071658114789 |
| D1 | 1.9098375817412905 | 0.23124846351870032 |
| D10 | 1.3510786515384818 | 2.5499320179613356 |
| D0 | 1.0859203414103817 | 0.35565609498395306 |
| D1 | 0.821463150008122 | 0.31457296025966097 |
| D10 | 1.2652676369843114 | 1.078835140688163 |
| D0 | 1.3677355408322789 | 0.3403804039653022 |
| D1 | 0.8755951447127496 | 0.2459841946556544 |
| D10 | 0.9326981290426718 | 0.2908575080237454 |
| D0 | 1.6404195529736187 | 0.5903702925641073 |
| D1 | 1.62429058015423 | 1.2918122710702267 |
| D10 | 0.6903359572618045 | 0.2918498643484764 |
| D0 | 1.8351239532776857 | 1.1160358940393693 |
| D1 | 1.6644872315562798 | 0.7454979914518728 |
| D10 | 0.40821852337438697 | 0.3616959592771223 |d0
d45
d2
d30
d4
d15
d8
*
*
*
Crenarchaeota
d0
d45
d2
d30
d4
d15
d8
*
*
*
*
Planctomycetes
*
*
P
SJF
d : day after treatment
D : treatment

## Slide 5
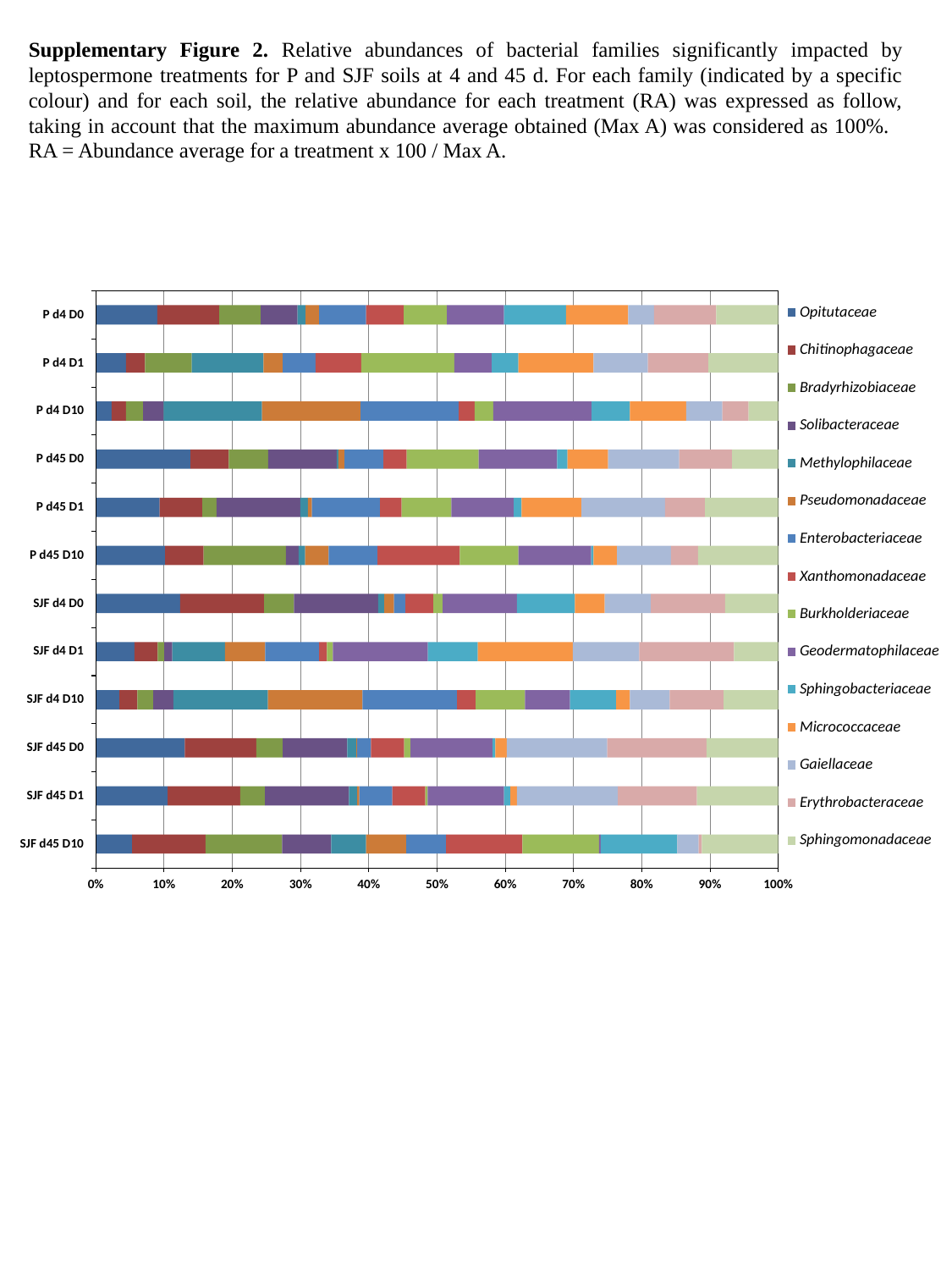

Supplementary Figure 2. Relative abundances of bacterial families significantly impacted by leptospermone treatments for P and SJF soils at 4 and 45 d. For each family (indicated by a specific colour) and for each soil, the relative abundance for each treatment (RA) was expressed as follow, taking in account that the maximum abundance average obtained (Max A) was considered as 100%. RA = Abundance average for a treatment x 100 / Max A.
